# Prevalence and associated factors of HIV-TB co-infection among HIV patients: a retrospective Study

**DOI:** 10.4314/ahs.v21i3.7

**Published:** 2021-09

**Authors:** Melkamu A Zeru

**Affiliations:** Department of Statistics, Bahir Dar University, Ethiopia

**Keywords:** Prevalence, TB, HIV, co-infection, Harari Region

## Abstract

**Background:**

TB/HIV co-infection is a major public health problem in many parts of the world. But the prevalence of co-infection was varies among countries.

This study was designed to assess prevalence of TB/HIV co-infection and to determine its factors.

**Methods:**

A retrospective study was done among HIV-positive patients at Hiwot Fana hospital from December, 2014 to 2018. The study participants were selected by simple random sampling. Patients with incomplete chart reviews were excluded and demographic, clinical and laboratory information were analyzed using SPSS and STATA. Uni-vitiate and bivariate logistic regressions were applied.

**Results:**

Five hundred fourteen patients were enrolled in this study. Of these, 187(37.4%) had TB. Bivariate logistic analysis showed that HIV patients with regards to marital status[AOR = 2.6; 95%CI = 1.19–2.89], education status [AOR = 3.74; 95%CI = 2.47–5.66], weight less than 50kg [AOR = 2.54; 95% CI = 1.35 – 4.81], CD4 level < 200cells/mm^3^ [AOR = 4.57; 95%CI = 2.38– 6.86] and patient who were at WHO clinical stage III [AOR = 7.8; 95%CI = 5.15 – 8.55] were significantly associated with TB/HIV co-infection.

**Conclusion:**

The prevalence of TB among HIV patients was high and predicted by marital, education status, weight, CD4 cell count and WHO clinical stage III.

## Introduction

TB and HIV co-infection is a major public health problem in many parts of the world. But the prevalence of TB/HIV co-infection was varies among countries. Exploring the reasons of the diversity of TB/HIV co-infection is important for public policy, planning and development of collaborative TB/HIV activities.^25^

Tuberculosis is the most common presenting opportunistic infection and a major public health problem. It is the cause of death among infectious diseases for people living with HIV/AIDS worldwide 1–3.

Worldwide, there were an estimated of 9 million incident cases of tuberculosis in 2013 with 13% co-infected with HIV. An estimated 1.5 million deaths were attributed to TB, excluding deaths among HIV-infected patients. Among infectious diseases, both tuberculosis (TB) and HIV/AIDS represent global public health emergencies. Their mutually detrimental effect on the individual patient and at the population level is most evident in sub-Saharan African countries 4.

According to the World Health Organization report Ethiopia ranked as the 7th country among the 22 high burden countries with TB - HIV infection in the world^5^. TB and HIV/AIDS display a lethal bidirectional interaction, with major epidemic overlap. People living with HIV are almost 30 times more likely than HIV-uninfected people to develop TB, and the chronic immune stimulation resulting from active TB accelerates HIV/AIDS disease progression 6–8.

Unlike other opportunistic infections, TB disproportionately affects people living with HIV even before any significant drop in CD4+ T cell counts. The key predictor of immune reconstitution inflammatory syndrome (IRIS) is the degree of HIV-induced immunosuppression (low CD4+ count) at ART initiation 9.

The current increasing of HIV/AIDS associated tuberculosis shifted the clinical pattern of TB towards smear negative pulmonary TB (PTB) and extra-pulmonary TB (EPTB), which in turn, causes difficulties in the diagnosis and treatment of TB due to unusual clinical picture with increased smear negative acid fast bacilli (AFB) PTB, atypical finding on chest radiography and increased prevalence of EPTB 10, 11.

Several studies confirmed that the clinical manifestations of TB in people with HIV are quite different and generally show different patterns as a function of the CD4+ T cell count. In addition, TB can appear at any stage of HIV/AIDS infection, and its presentation varies with the WHO stage and CD4+ lymphocyte count, although it is more frequent when CD4+ T cell count is below 200 cells/mm^3^ 12. Research findings indicated that some HIV-infectd people develop TB, while others do not. This idea implies that being HIV positive is not the only factor for being infected with TB, and there are various determinant factors that contribute to TB/ HIV co-infection 12–14. Understanding the predictors of TB/HIV co-infections in the local context is critical point for Ethiopia to improve TB/HIV co-infected patients. In the study area, there were no relevant research done on the prevalence and determinants of TB-HIV co-infection.

The objective of this study was to determine the prevalence and factors associated with TB co-infection among HIV patients in Hiwot Fana hospital in Harar region, which will help to reduce the risk of death and its burden by taking early drug management in the local context.

## Methods

### Case Definitions

The TB case definitions below are based on the level of certainty of the diagnosis and on whether or not laboratory confirmation is:

Tuberculosis suspect. Any person who presents with symptoms or signs suggestive of TB. The most common symptom of pulmonary TB is a productive cough for more than 2 weeks.

Case of tuberculosis. A definite case of TB in which a health worker (clinician or other medical practitioner) has diagnosed TB and has decided to treat the patient with a full course of TB treatment. Any person given treatment for TB should be recorded as a case. Incomplete trial TB treatment should not be given as a method for diagnosis.

Definite case of tuberculosis. A patient with Mycobacterium tuberculosis complex identified from a clinical specimen, either by culture or by a newer method such as molecular line probe assay.

### Study Design and Area

This study was a hospital based retrospective study addressing the prevalence of TB among HIV/AIDS. It used data from the HIV patients attending the ART clinic from December, 2014 to December, 2018 in Hiwot Fana hospital in Harari region, Ethiopia. Hiwot Fana Specialized University hospital serves as a referral hospital for the entire Eastern part of Ethiopia, including Eastern Oromia, Dire Dawa city administration, the Somali regional state and the Harari regional state. The facility is treats more than 2,097 HIV positive patients. It is a teaching hospital for Haramaya University health and medical sciences students. Hiwot Fana specialized University hospital obstetric department admits more than 3000 patients per year, with 2664 deliveries.

### Sample size and sampling procedure

The sample size was determined by taking the estimated proportion (P) of TB15 among people with HIV positive ratio= 0.1, margin of error = 2 % and confidence level = 95%, and the required sample size became 864. The 4 years HIV/AIDS patients' case files were identified and the study participants were selected by simple random sampling which gives an equal chance for each subjects to be included in the study, since the nature of target population is heterogeneous, to increase the precision on the study findings. After excluding patients with incomplete medical records, and by adding 10 % to replace the excluded samples, 514 HIV patients who were under ART were considered as study samples to collect the data.

### Data collection instruments and procedures

Data were collected from patients' chart files using a checklist which was prepared by adopting previous studies 16–18. Pre-test was conducted for the check list before the actual data collection and some variables such as religion, employment status, functional status and alcohol intake were omitted because of incomplete medical records. Otherwise, the checklist was inclusive of the different socio-demographic variables (sex, age, education status, marital status, residence) and clinical characteristics (CD4 count, weight) which were assumed as possible determinants of TB occurrence among people with HIV. Data quality was assured by pre-tested data collection and by trained data collectors. Completeness and consistency of data was checked by professionals and data clerks before and after data entry.

### Data Analysis

The data was collected and entered in-to the statistical package for social science (SPSS) version 23 and STATA version 14 were used. Frequency and percentage were determined for descriptive analysis of socio-demographic, clinical and laboratory profile of the study subjects. Multivariable logistic regression was used by entering all variables with p-value < 0.25 in the bivariate analysis. Finally, logistic regression with forward likelihood variable selection method with p-value < 0.05 was used to identify the determinant factors associated with TB/HIV co-infection. The Hosmer-Lemeshow test of goodness of fit was used to model diagnosis with p-value > 0.05 provided good fit for the model.

## Results

### Socio-demographic characteristics

Medical records chart of 514 people living with HIV were used. The mean age of the study participants was 34.17 years with standard deviation (SD) of 3.6 years (34.71±3.6). Most of the participants were females 270(52.5%), aged greater than or equal 30 years were 296(57.6%), living in urban residents were 273(53.2%). Moreover, 282(54.9%) of the study participants were married and 310(60.3%) of the participant were educated (Table 2).

**Table 2:** Socio-demographic characteristics of study participants in Hiwot Fana Referral Hospital (n = 514)

Variables	Frequency	Percent (%)
Sex		
Male	244	47.5
Female	270	52.5
Age		
<30 years	21	42.4
≥ 30 year	296	57.6
Marital status		
Married	282	54.9
Others	232	45.11
Education status		
Illiterate	204	39.7
Literate	310	60.3
Residence		
Rural	241	46.9
Urban	273	53.2

There were 514 participants in the study. Of these 187(37.4%) of them were had HIV/TB co-infection. Of the total, 187 TB/HIV co-infected, female respondents had higher proportion 108(57.8%) as compared to male respondents 79(42.2%). Patients whose CD4 count was less than 200cells/mm^3^ had larger proportion 120 (64.4%) as compared to those CD4 count ≥ 200 cells/mm^3^ 67(35.6%). Furthermore, majority of TB/HIV co-infected patients 107 (57.2%) live in urban residence. From the same result among TB/HIV patients 95(50.8%) were married while 92(49.2%) were others (single, divorced and widowed). Moreover, out of TB/HIV co-infected patients, 76(40.6%), were found in WHO clinical stage of III, 73(39%) in WHO clinical stage of IV, 21(11.2%) under WHO clinical stage of III and 17(9.1%) in WHO clinical stage of I. In addition, more than half of co-infected patients 98(52.4%) but the remaining were illiterates (Table 3).

**Table 3:** Bivariate and Multivariable association of TB/HIV co-infection and independent factors

	HIV/TB Co-infected					

	Yes	No	COR (95%CI)	P-value	AOR (95% CI)	P-value
Sex						
Male	79	165	0.72(0.49 – 1.03)	0.34	0.78(1.31 – 2.96)	0.230
Female	108	162	1		1	
Age						
< 30 year	93	125	0.62(0.43–0.89)	0.15	0.52(3.34 – 5.79)	0.200
≥ 30 year	94	202	1		1	
Marital status						
Married	95	137	1		1	
Others	92	190	1.69(1.48 – 2.01)	0.042*	2.6(1.19–2.89)	0.011
Education status						
Illiterate	98	106	3.06(2.10–4.44)	0.01*		
Literate	81	229	1			
Residence						
Rural	80	183	1.69(1.18 – 2.44)	0.2	1.97(1.31 – 2.96)	0.110
Urban	107	144	1		1	
Weight						
≤ 50 kg	94	129	0.64(0.44–0.92)	0.01*	2.54(1.35 – 4.81)	0.003
>50 kg	93	98	1		1	
CD4 Count						
<200cells/m^3^	120	165	1.55(2.38 – 4.79)	0.002*	4.57(2.38 – 6.86)	0.007
≥200cells/mm^3^	67	162	1		1	*
Clinical stage						
Stage I	17	33	1		1	
Stage II	21	87	1.49(0.97 – 2.28)	0.12	1.27(0.79 – 2.03)	0.310
Stage III	76	85	0.403(0.23 – 0.70)	0.04*	7.8(5.15 – 8.55)	0.000
Stage IV	73	122	0.86(0.44 – 1.65)	0.28	0.29(0.38 – 1.59)	0.500

Hosmer - Lemenshow test p-value		0.673*				

* means statistically significant at 5 % level of significance (p-value < 0.05)

Others means(Single, divorced and Windowed)

The Hosmer-Lemeshow test provided good fit for the model (p-value =0.673)

Results in the bivariate analysis showed that the variable residence, WHO clinical stage, education status, marital status, CD4 counts and weight were associated with TB/HIV co-infection. Multivariate analysis in multiple logistic regression was used to determine factors of TB/HIV co-infection after adjusting other covariates. From this result, HIV patients whose marital status was different from married were 2.6 times more likely to develop TB than that of married [AOR= 2.6; 95 % CI = 1.19 – 2.89]. The illiterate patients were 3.74 times more likely to develop TB as compared to the educated patients [AOR = 3.74; 95% CI = 2.47 – 5.66]. Furthermore, patients whose CD4 cell level < 200 cell/mm^3^ were 4.57 times more likely to develop TB as compared with cell level ≥ 200 cell/mm3 [AOR = 4.57; 95% CI = 2.38 – 6.86]. This implies low CD4 cell count is potential determinant for TB/HIV co-infection. Similarly, patients in WHO clinical stage III were 7.8 times more likely to develop TB as compared to that of patient in WHO clinical stage I[AOR = 7.8; 95%CI = 5.15 – 8.55] (Table 3).

## Discussion

Tuberculosis is an opportunistic infection in patients with HIV. The prevalence of TB/HIV co-infection in this study was 37.4%. This in line studies in southern Ethiopia (36.9%) 19, in Addis Ababa (33%) 20 and the study conducted in Keniya (35.6 %) 21. However, the finding of this study was higher compared to the study result in Amhara region, Ethiopia (27.7%) 12 and a study reported from Nigeria (11%) 22. The possible reasons for this large variation in TB/HIV co-infection across the world may be due to difference in study settings, sample size, diagnosis facility, HIV infection rate, study area and study time.

Being at WHO clinical stage III, having a CD4 level < 200 cells/mm^3^, were identified as risk factors for TB development among HIV patients. These finding is consistent with other Hospital-based studies in Ethiopia 12, 19 and the study in Nigeria 23. In my study patients with marital status (single, divorced and widowed) were morelikely to develop TB, which is in line with the study of 12 Mitku and others. The literate were less likely to develop TB. This finding is consistent with the results in Ethiopia 22 and Zambia 24. In contrast, a study in western Ethiopia 12 and in Addis Ababa, confirms that high level of education was associated with TB. The contradiction may be possibly due to large proportion TB/HIV co-infection among illiterate patients in my study area.

## Conclusion

In this study the prevalence of TB/HIV co-infection was high (37.4%). The risk of TB/HIV occurrence was found to be high among patients whose marital status being single, divorced & widowed, who was illiterate, whose weight was < 50kg, CD4 level were below 200celsl/mm^3^, and patients in WHO clinical stage III. The large ratio of TB cases among HIV patients requires a special attention for clinical and radiological investigation. Furthermore, since clinical variables were associated with TB/HIV co-infection, health professionals should care for patients who are in WHO clinical stage III, and patient who had lower CD4 counts.

## Limitation of the study

Even though I endeavored to identify factors associated with TB/HIV co infection, the study was conducted in a single hospital with small number of patients, not include variables like viral load, TB before ART, sputum positivity and others, it is difficult to make generalizations about the population and community-based studies are needed to substantiate the study conclusions.

## Figures and Tables

**Table 1:** Variables used in the Study

S/N	Variable	Description
1.	Sex	0 = Female, 1= Male
2.	Age	0 = < 30 year 1 = 1 ≥ 30 year
3.	Marital status	0 = Married, 1 = Others
4.	Education status	0 = Illiterate , 1 = Literate
5.	Residence	0 = Rural , 1 = Urban
6.	Weight	0 = ≤ 50 kg, 1 = >50 kg
7.	CD4 Count	0 = < 200 cells/mm^3^, 1 = ≥200 cells/mm^3^
8.	Clinical stage	0 = Stage I, 1 = Stage II 2, = Stage III, 3 = Stage IV

## Data Availability

All data generated or analyzed during this study are available from the corresponding author on reasonable request.

## Abbreviations

AFT: Acid Fast Bacilli; AOR: Adjusted Odds Ratio; CI: Confidence Interval; COR: Crude Odds Ratio; EPTB: Extra Pulmonary Tuberculosis; HIV: Human Immunodeficiency Virus; PTB: pulmonary Tuberculosis; TB: Tuberculosis; WHO: World Health Organization
